# Prognostic value of peritoneal scar-like tissue in patients with peritoneal metastases of ovarian origin presenting for curative-intent cytoreductive surgery

**DOI:** 10.1186/s12957-023-03153-z

**Published:** 2023-08-28

**Authors:** Antoine El Asmar, Florin Pop, Etienne El Helou, Pieter Demetter, Isabelle Veys, Laura Polastro, Ali Bohlok, Gabriel Liberale

**Affiliations:** 1 Department of Surgical Oncology, Institut Jules Bordet, Université Libre de Bruxelles, 90 Rue Meylemeersch, 1070 Brussels, Belgium; 2 Department of Pathology, Institut Jules Bordet, Université Libre de Bruxelles, Brussels, Belgium; 3 Department of Medical Oncology, Institut Jules Bordet, Université Libre de Bruxelles, Brussels, Belgium

**Keywords:** Ovarian cancer, Peritoneal carcinomatosis, Peritoneal fibroses, Cytoreductive surgery, Prognostic factors

## Abstract

**Background:**

Complete cytoreductive surgery (CRS), remain the gold standard in the treatment of peritoneal metastases of ovarian cancer (PMOC). Given the increasing rate of neoadjuvant chemotherapy in patients with high PCI, prior abdominal surgeries, inflammation and fibrotic changes, the benefit of removing any “peritoneal scar-like tissues” (PST) during CRS, hasn’t been thoroughly investigated. Our objective in this retrospective cohort was to identify the proportion of malignant cells positivity in PST of patients with PMOC, undergoing curative-intent CRS ± HIPEC.

**Methods:**

This is a retrospective study, conducted at our comprehensive cancer center, including patients with PMOC, presenting for curative-intent CRS. During CRS, benign-looking peritoneal lesions, lacking the typical hard nodular, aggressive, and invasive morphology, were systematically resected or electro fulgurated. PSTs were analyzed for the presence of tumoral cells by our pathologist. Correlations between the presence of PST and their positivity, and the different patients’ variables, were studied.

**Results:**

In 51% of patients, PST harbored malignant cells. Those were associated with poorly differentiated serous tumors, a high PCI (> 8) and a worse DFS: 17 months in the *positive PST group* versus 29 months in the *negative PST group* (*p* = *0.05*), on univariate analysis. Multivariate analysis revealed that PCI > 8 and poorly differentiated primary tumor histology were correlated with a worse DFS, and that higher PCI and advanced FIGO were correlated with a worse OS.

**Conclusion:**

Benign-looking PST harbors malignancy in 51% of cases. The benefit of their systematic resection and their prognostic value should be further investigated in larger cohorts.

## Introduction

Ovarian cancer (OC) is the most common cause of cancer-associated death in women, and is responsible for approximately 150,000 annual deaths worldwide [1, 2]. Around 75% present with advanced stages and a 5-year survival rate less than 50% [3]. The peritoneal carcinomatosis index (PCI) and complete cytoreductive surgery (CRS) remain the most important prognostic factors in determining the survival of these patients [4]. Attempts to downstage patients with a very high peritoneal disease burden prior to surgery, using neoadjuvant chemotherapy (NACT), revealed successful in decreasing surgery-related morbidities, by decreasing the extent of resections required to achieve complete cytoreduction (CCR-0) and achieving similar results to upfront surgery [5]. Macroscopic complete cytoreduction (CCR-0) remains the pillar on which surgeons should rely in order to achieve the best patients’ outcome [6].

Currently, intraoperative detection of tumor lesions involves palpation and visual inspection. Palpation limits the surgeon’s ability to identify non-palpable flat lesions, while visual inspection difficulty lies in poor tissue contrast and spatial resolution [7]. Multiple studies attempted quantifying residual tumors in patients with PMOC, however, with the use of NACT, especially in cases with a high PCI, microscopically carcinomatous areas can present with a benign visual appearance. This leads to an underestimation of the tumoral spread, and hence potential incomplete resections [2, 8]. This has been reported as an argument by upfront protagonists as it allows resecting all visible disease before neoadjuvant treatment. However, the counter part is the lower rate of CCR-0 resection [9].

A major concern in patients treated by NACT for peritoneal metastases of OC origin (PMOC) is the management of residual scar-like lesions. Moreover, many PMOC patients, who did not receive NACT, can present with scar-like lesions on their peritoneum, either due to previous surgeries, local inflammatory reactions, granulomas or fibrosis, or benign lesions. These peritoneal patches usually lack the typical nodular, granular, invasive morphology that PM have, and thus are usually considered benign and not subject to resection. At present, no clear rules exist concerning the intraoperative management of these lesions. Very few study have focused on the pathology of peritoneal scars and their prognostic role remains undetermined [2, 7, 9, 10].

More recently, intraoperative fluorescence imaging (FI) techniques have been used trying to increase residual tumor detection in ovarian cancer [10]. van Dam et al. has reported encouraging results in a pilot study on folate-receptor-α positive ovarian cancer patients, using specific FI technique [11]. Moreover, Veys et al. have reported the role of ICG-FI in detecting residual peritoneal disease. They were able to detect malignant cells in 68% of resected peritoneal scar tissue in patients with PMOC. ICG-FI revealed to be an accurate tool in identifying PM, however, given its relatively low positive predictive value of 57%, it was not able to discriminate between benign and malignant lesions post-NACT [10].

The objective of this study was to identify the proportion of malignant cells positivity in peritoneal scar-like tissue (PST), in patients with PMOC, who underwent or not NACT, to evaluate if there are predictive factors for the presence of tumoral scars, and analyze the potential prognostic value of positive PST.

## Patients and methods

### Study design and population

This is a retrospective study, conducted at our comprehensive cancer center, Institut Jules Bordet (Université Libre de Bruxelles – part of the “Hopitaux Universitaires de Bruxelles”, H.U.B.) including patients with PMOC, presenting for curative-intent CRS ± HIPEC, from 2012 until 2018. The study was approved by the ethical committee at Institut Jules Bordet (CE3375).

### Peritoneal scar-like tissue

During CRS, benign-looking peritoneal lesions are systematically resected or electro fulgurated in all patients with PMOC. Visual and tactile inspection was undergone by the surgeon in order to categorize these lesions as “scar-like tissue” rather than “peritoneal metastases”, lacking the typical hard nodular, aggressive, and invasive morphology.

Peritoneal scar-like tissue (PST) was defined using the following visual and tactile characteristics:

- Flat demarcated zone of the visceral or parietal peritoneum,
- Whitish discoloration,
- Absence of nodules or nodular formation,
- Soft, non-granular on palpation
- Flaccid, loose, non-rigid upon resection.

**Fig. 1:**
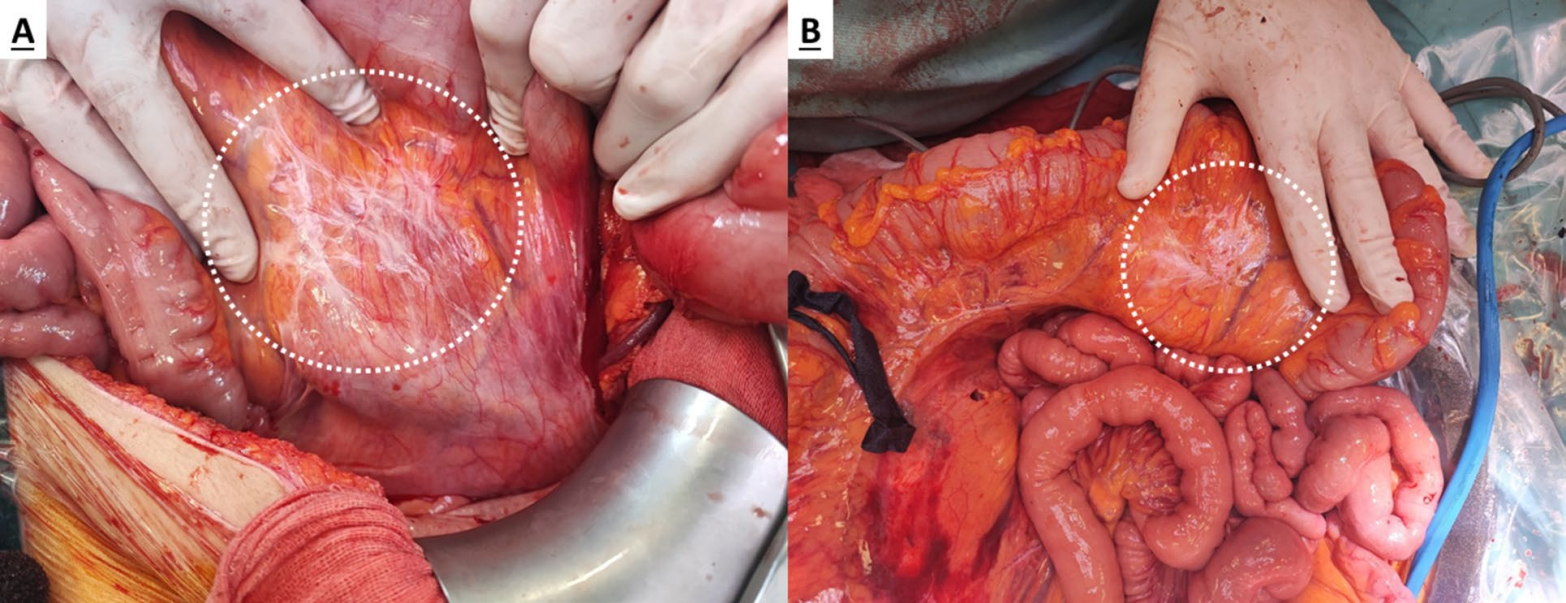
Benign peritoneal scar-like tissue (**A**) versus malignant peritoneal scar-like tissue (**B**), as confirmed by pathology examination, on the mesocolon of a patient presenting with PMOC

These areas consisted mainly of residual scar tissue post-NACT, exhibiting partial or complete pathological response, or areas of postoperative adhesions, or fibrosis, or granulomas, in patients who did not undergo NACT prior to the CRS (Fig. 1). Typical lymphatic serosal discoloration on the small bowel or on the Glisson capsule were not included in this definition.

### Histopathological analysis

All resected PSTs were sent to histopathological analysis. Upon inspection, the pathologist reported the presence or absence of malignant cells within each PST.

### Statistical analysis

A descriptive analysis of the whole population was conducted. Pre-, intra-, and post-operative clinical, demographical and histopathological patients’ characteristics were included. The proportion of *PST presence* and *PST positivity* for malignant cells was calculated. Values are expressed as medians (interquartile range, IQR), means (standard deviations, SD), or the number of patients with percentages. Medians and means were compared using the Mann–Whitney U test and the Student’s t-test, respectively. Differences in proportions were evaluated using the chi-square test. We performed univariate and multivariate analyses to evaluate the correlations, first between the presence of PST and the different variables, and second between the positivity of PST and the different variables. Risk factors with a *p* < *0.1* were entered into the multivariate model. Disease-free survival (DFS) and overall survival (OS) were calculated for the whole population and for each group of patients separately (PST positive vs PST negative patients’ categories) using the Kaplan Meier method and the statistical significance was calculated using the log-rank test. The factors affecting survival were evaluated using a univariate and multivariate cox regression analysis. Proportional hazard regression results are reported using hazard ratios (HR) and corresponding 95% confidence intervals (Cis). Factors with a *p* value of < 0.05 in univariate analysis were entered to a multivariate cox regression model. A *p* < *0.05* was considered statistically significant. Statistical analyses were performed using SPSS VERSION 28.

## Results

During the study period, 193 patients with PMOC underwent curative-intent CRS at our institution. The mean population age was 61 years, 69% had NACT for locally advanced disease, 12% were subject to HIPEC and the median preoperative PCI was 8. The majority presented with high grade serous ovarian cancer. An CCR-0 resection was achieved in 176 patients (91.2%), while 17 patients (8.8%) were subject to an R2a resection. On final pathology reports, 80% of patients were classified in the FIGO III category, versus 14% FIGO IVa and 6% FIGO II. The median DFS and OS for the whole population were 22 and 66 months respectively. The median follow-up time was 82 months (70.9–93.1). Patients characteristics are reported in Table 1.

**Table 1:** Patients’ pre-, intra-, and post-operative demographic, clinical and histopathological characteristics

Variables	*N* = 193 (%)	Absence of PST *N* = 114 (59.1%)^c^	Presence of PST *N* = 79 (40.9%)^c^	*p*	Negative PST *N* = 39 (49.3%)^c^	Positive PST *N* = 40 (50.7%)^c^	*p*
Mean Age (± SD) in years	61 (± 12)	58.8 (± 11.9)	63.7 (± 11.7)	***0.005***	62.5 (12)	64.8 (11.4)	*0.39*
Median BMI [IQR] kg/m^2^	23.8 [6]	23.8 [7]	23.8 [6]	*0.91*	24 [5]	23.6 [7]	*0.91*
ASA score							
I	11 (5.7)	7 (6.1)	4 (5.1)	*0.37*	2 (5.2)	2 (5)	*0.91*
II	138 (71.5)	85 (74.6)	53 (67.1)		27 (69.2)	26 (65)	
III	44 (22.8)	22 (19.3)	22 (27.8)		10 (25.6)	12 (30)	
IV	-	-	-		-	-	
Median CA125 pre-NAC [IQR] u/ml	321 [820]	367 [877.5]	257 [803]	*0.26*	522 [1118]	210 [256.7]	*0.58*
Median CA125 post-NAC [IQR] u/ml	38 [139.5]	42.5 [210.7]	30 [87]	*0.59*	31 [142]	29 [60]	*0.91*
NAC							
Yes	133 (69)	76 (66.7)	57 (72.2)	*0.41*	29 (74.4)	28 (70)	*0.66*
No	60 (31)	38 (33.3)	22 (27.8)		10 (25.6)	12 (30)	
Median number of NAC cycles	3 [4]	3 [4]	3 [4]	*0.94*	3 [4]	3 [4]	*0.75*
FIGO stage^a^							
II	12 (6.2)	9 (7.9)	3 (3.8)	*0.48*	3 (7.7)	0	*0.20*
III	153 (79.3)	88 (77.2)	65 (82.3)		31 (79.5)	34 (85)	
IVa^b^	28 (14.5)	17 (14.9)	11 (13.9)		5 (12.8)	6 (15)	
Primary tumor histology							
Serous	169 (87.5)	100 (87.7)	69 (87.3)	*0.93*	31 (79.5)	38 (95)	***0.038***
Other or mixed	24 (12.5)	14 (12.3)	10 (12.7)		8 (20.5)	2 (5)	
Degree of differentiation							
Well	27 (14)	15 (13.2)	12 (15.2)	*0.66*	10 (25.7)	2 (5)	***0.028***
Moderate	27 (14)	18 (15.8)	9 (11.4)		5 (12.8)	4 (10)	
Poor	139 (72)	81 (71)	58 (73.4)		24 (61.5)	34 (85)	
Lymph nodes status							
Positive	53 (27.6)	83 (72.8)	56 (71.8)	*0.88*	13 (34.2)	9 (22.5)	*0.25*
Negative	139 (72.4)	31 (27.2)	22 (28.2)		25 (65.8)	31 (77.5)	
Median PCI [IQR]	8 [12]	9 [12]	7 [11]	*0.38*	5 [10]	9 [13]	***0.03***
HIPEC							
No	170 (88.1)	107 (93.9)	63 (79.7)	***0.003***	30 (76.9)	33 (82.5)	*0.53*
Yes	23 (11.9)	7 (6.1)	16 (20.3)		9 (23.1)	7 (17.5)	
Median intraoperative blood loss [IQR] in cc	1300 [1993]	1000 [1800]	1600 [2025]	***0.043***	1825 (2075)	1500 [1900]	*0.39*
Median CRS time [IQR] in min	326 [275]	283 [264]	390 [208]	***0.001***	406 [206]	380 [216]	*0.12*
CD complications	191	113	78	*0.21*	38	40	*0.65*
No complications	86 (45)	56 (49.5)	30 (38.5)		17 (44.7)	13 (32.5)	
CD-I	31 (16.2)	19 (16.8)	12 (15.4)		5 (13.2)	7 (17.5)	
CD-II	49 (25.7)	23 (20.4)	26 (33.3)		13 (34.2)	13 (32.5)	
CD-IIIA	7 (3.7)	3 (2.7)	4 (5.1)		2 (5.3)	2 (5)	
CD-IIIB	7 (3.7)	4 (3.5)	3 (3.8)		1 (2.6)	2 (5)	
CD-IV	10 (5.2)	8 (7.1)	2 (2.6)		0	2 (5)	
CD-V	1 (0.5)	0	1 (1.3)		0	1 (2.5)	
Median number of scar [IQR]	-	-	2 [2]	*-*	2 [2]	2 [2]	*0.94*
Median DFS (95% CI) in months	22 (18.8–25.2)	22 (16.8–27.1)	22 (16.8–27.1)	*0.88*	29 (20.1–37.9)	17 (14.9–19)	***0.05***
Median OS (95% CI) in months	66 (58.9–73.1)	64 (49.8–78.1)	66 (56.3–75.6)	*0.56*	68 (43.2–92.8)	64 (48.4–79.6)	*0.63*

^a^FIGO stage reported on final pathology

^b^Patients with FIGO IVa were included, with resectable peritoneal disease, and in whom CRS was complete

^c^Relative Risk within the concerned category

*PST* Peritoneal Scar-like Tissue, *SD* Standard deviation, *IQR* Interquartile range, *BMI* Body mass index, *PCI* Peritoneal carcinomatosis index, *ASA* American Society of Anesthesiologists, *NAC* Neoadjuvant chemotherapy, pre-cytoreductive surgery, *HIPEC* Hyperthermic intraperitoneal chemotherapy, *CRS* Cytoreductive surgery, *CD* Clavien-Dindo

### Peritoneal scar-like tissue

Peritoneal scar-like tissue was found in 79 patients (40.9%). PSTs were harboring malignant cells in 40 patients (50.7%). Positive PSTs were associated with serous tumors, poorly differentiated, a high PCI (> 8) and a worse DFS: 17 months in the *positive PST group* versus 29 months in the *negative PST group* (*p* = *0.05*). However, none of these factors were independent predictors of PST positivity on multivariate analysis (Table 1). In the group of patients *with PST*, 57 (72%) were subject to NACT and 22 (28%) were not. In addition, amongst patients who received NACT, 28 (70%) had PST positive for tumoral cells, versus 12 (30%) patients with positive PST, amongst those who did not receive any neoadjuvant treatment.

In terms of prognosis, an advanced FIGO stage, a PCI > 8, and poorly differentiated tumor histology were also associated with a worse DFS (HR = 2.62, 95% CI: 1.15–5.97, *p* = *0.02;* HR = 1.97, 95% CI: 1.41–2.76, *p* < *0.001*; HR = 2.2, 95% CI: 1.15–4.22, *p* = *0.01*, respectively) on univariate analysis. Multivariate analysis revealed that PCI > 8, and poorly differentiated primary tumor histology, were significant independent variables correlated with an unfavorable DFS. Furthermore, only higher PCI (HR = 1.87, 95% CI: 1.2–2.9), and an advanced FIGO stage (HR = 3.4, 95% CI: 0.9–12.2) were predictors of worse OS on multivariate analysis (Table 2).

**Table 2:** Univariate and Multivariate analyses of prognostic factors in patients with PMOC presenting for curative-intent CRS

Variables	DFS				OS			
	**Univariate**		**Mutlivariate**		**Univariate**		**Mutlivariate**	
	HR (95%CI)	*p*	HR (95%CI)	*p*	HR (95%CI)	*p*	HR (95%CI)	*p*
Age (cont)	1.01 (0.99–1.02)	*0.35*			1.01 (0.99–1.03)	*0.19*		
BMI (cont)	1.00 (0.97–1.03)	*0.91*			1.01 (0.97–1.05)	*0.76*		
CA125 pre-Tx > 321 u/ml	1.27 (0.91–1.77)	*0.16*			1.25 (0.85–1.85)	*0.25*		
CA 125 post-Tx > 38 u/ml	1.11 (0.80–1.55)	*0.51*			1.39 (0.94–2.07)	*0.09*	1.13 (0.74–1.74)	*0.57*
NAC	0.88 (0.62–1.25)	*0.47*			0.93 (0.62–1.39)	*0.73*		
FIGO								
III vs II	2.62 (1.15-	***0.02***	1.81 (0.78-	*0.17*	3.73 (1.17-	***0.025***	2.52 (0.76-	*0.13*
IVa vs II	5.97) 3.01 (1.21–7.44)	*0.17*	4.24) 1.88 (0.73–4.82)	*0.19*	11.8) 5.35 (1.56–18.3)	***0.008***	8.31) 3.43 (0.96–12.27)	***0.05***
PCI > 8	1.97 (1.41–2.76)	** < *0.001***	1.78 (1.25–2.53)	***0.001***	2.16 (1.45–3.24)	** < *0.001***	1.87 (1.20–2.91)	***0.005***
Primary tumor histology:								
Serous vs Other or mixed	0.76 (0.44–1.31)	*0.32*			0.92 (0.50–1.69)	*0.79*		
Degree of differentiation:								
Moderate vs well	2.04 (0.96–4.32)	*0.06*	2.04 (0.96–4.32)	*0.06*	2.51 (1.11–5.68)	***0.027***	2.19 (0.95–5.04)	*0.06*
Poor vs well	2.20 (1.15–4.22)	***0.01***	2.03 (1.06–3.89)	***0.03***	1.84 (0.89–3.83)	*0.099*	1.53 (0.73–2.21)	*0.26*
Positive LN status	0.96 (0.67–1.38)	*0.86*			0.99 (0.65–1.50)	*0.97*		
HIPEC	0.83 (0.47–1.46)	*0.52*			0.86 (0.44–1.65)	*0.65*		
CD complication:								
CD 0-I-II vs CD III-IV	1.14 (0.71–1.83)	*0.59*			1.19 (0.69–2.03)	*0.52*		
Positive PST	1.35 (0.92–1.99)	*0.12*	1.15 (0.77–1.70)	*0.49*	1.01 (0.62–1.61)	*0.97*		

*BMI* Body mass index, *PCI* pEritoneal carcinomatosis index, *NAC* Neoadjuvant chemotherapy, pre-cytoreductive surgery, *LN* Lymph nodes, *HIPEC*: Hyperthermic intraperitoneal chemotherapy, *CRS* Cytoreductive surgery, *CD* Clavien-Dindo, *PST* Peritoneal scar tissue

## Discussion

We hereby offer one of the first studies evaluating residual disease in peritoneal scars in patients treated by CRS ± HIPEC for ovarian cancer peritoneal metastases. We observed that around 51% of patients had microscopic residual disease in resected peritoneal scar-like lesions. The presence of positive peritoneal scar-like lesions were significantly associated with worse DFS on univariate analysis, 17 months for positive versus 29 months for negative PST.

Given the need for new prognostic factors to optimize PMOC patients’ selection and management, every clinical, histopathological and anatomical aspect, capable of enhancing the outcome of curative-intent CRS, should to be further explored.

PM from OC origin have a less aggressive behavior compared to those from colorectal or gastric origin, reason why patients with a relatively higher PCI are still eligible for surgery [12, 13]. This warrants a higher rate of gastrointestinal resections, splenectomies and peritonectomies in PMOC patients compared to others. Platine-based chemotherapy and complete cytoreductive surgery, upfront or interval, remain the pillars of treatment in patients with PMOC [4, 14]. Major prognostic factors remain the radicality of the CRS, PCI score, tumor histology and platinum-sensitivity, along with ascites and certain molecular expressions in ongoing investigations [15–17]. This is corroborated by our study, reporting poorly differentiated tumors, advanced FIGO stage and high PCI score as independent prognostic factors in patients with PMOC.

Very few studies focused on the possible role of residual tumoral cells in peritoneal scar-like tissue [2, 10, 18]. Recently, we have reported the potential role of ICG-FI in detecting residual tumoral disease in peritoneal scars [10]. Despite the fact that ICG-FI was able to detect tumoral cells with a sensitivity of 73%, the reported specificity was low (57%).

The present study was aiming to analyze the rate and positivity of residual peritoneal scar-like tissue at pathology. It shows that benign-looking positive PST predict a worse median DFS (17 versus 29 months) on univariate analysis. However, it was not significant on multivariate analysis. Furthermore, we were not able to determine predictive factors for these PST positivity, especially when considering the group of patients with NACT versus those who did not take any neoadjuvant treatment. PST presence and positivity was not related to whether the patients received or not any type of NACT. This raises the question on whether PST should be systematically resected during CRS. At this stage, we still are unable to answer this question, and further studies on the effect of removing these PST are warranted. Many studies have proved an oncological benefit in performing an omentectomy of grossly normal omentum in patients presenting with ovarian cancer. Whether such routine could be extrapolated to PST in the future is still yet to be established.

Our study has numerous weaknesses and biases, starting with its retrospective design. In practice, the results of this study are based on our surgical attitude tending towards systematic resection or electrofulguration of all PST. However, some patients in the early stages of the study period were not operated while following this strategy. Therefore, it is possible that the rate of PST is still underestimated. This could also explain the absence of significant results for positive PST at multivariate analysis, given that a subgroup of patients is still harboring positive scar-like tissue. In addition, an operator-dependent bias is also present, given that visual and tactile evaluations were performed by the surgeon and his fellow. However, we tended to categorize lesions as “PST” using a set of morphological characteristics devised by the oncologic surgeons.

Still the results reported hereby, could constitute a modest foundation for further investigations in prospective studies. Moreover, the use of specific FI guided surgery in the future, should help the surgeons in detecting residual disease on a molecular level.

## Conclusion

Benign-looking PST harbor cancerous cells in 51% of cases, and could represent a prognostic factor in patients with PMOC. A large prospective study on the role of PST in patients with PMOC, presenting for curative-intent cytoreductive surgery, to evaluate their exact prognostic value and the benefit of their resection, could respond to these questions.

## Data Availability

Research data supporting this publication is available upon Editor’s request.
